# Clusters of Temporal Discordances Reveal Distinct Embryonic Patterning Mechanisms in *Drosophila* and *Anopheles*

**DOI:** 10.1371/journal.pbio.1000584

**Published:** 2011-01-25

**Authors:** Dmitri Papatsenko, Michael Levine, Yury Goltsev

**Affiliations:** Department of Molecular and Cell Biology, Division of Genetics Genomics and Development, Center for Integrative Genomics, University of California, Berkeley, California, United States of America; Harvard University, United States of America

## Abstract

Evolutionary innovations can be driven by spatial and temporal changes in gene expression. Several such differences have been documented in the embryos of lower and higher Diptera. One example is the reduction of the ancient extraembryonic envelope composed of amnion and serosa as seen in mosquitoes to the single amnioserosa of fruit flies. We used transcriptional datasets collected during the embryonic development of the fruit fly, *Drosophila melanogaster*, and the malaria mosquito, *Anopheles gambiae*, to search for whole-genome changes in gene expression underlying differences in their respective embryonic morphologies. We found that many orthologous gene pairs could be clustered based on the presence of coincident discordances in their temporal expression profiles. One such cluster contained genes expressed specifically in the mosquito serosa. As shown previously, this cluster is redeployed later in development at the time of cuticle synthesis. In addition, there is a striking difference in the temporal expression of a subset of maternal genes. Specifically, maternal transcripts that exhibit a sharp reduction at the time of the maternal-zygotic transition in Drosophila display sustained expression in the Anopheles embryo. We propose that gene clustering by local temporal discordance can be used for the *de novo* identification of the gene batteries underlying morphological diversity.

## Abstract 2

Evolving organs are seen as clusters of discordant genes on the heatmaps representing cross-species comparisons of developmental gene expression data.

## Author Summary

Linking genotype to phenotype is a major undertaking in modern biological research. A variety of strategies are used but have generally failed to explain the maintenance and acquisition of new phenotypic traits in changing populations. We propose that whole-genome cross-species comparisons can be used to identify gene clusters underlying phenotypic variation. In the present study we used gene expression datasets collected during fruit fly and mosquito embryogenesis to identify temporal changes in gene expression. We found that differentially represented tissue types (such as extraembryonic serosa) were clearly manifested by clusters of local discordances in gene expression. Discordances were also observed for a suite of maternally expressed genes, consistent with the notion that the abrupt maternal-zygotic transition seen in Drosophila is an evolutionary innovation of higher Diptera. We propose that gene clustering by expression discordance can be used to determine the genetic basis of phenotypic variation.

## Introduction

During the 1980s and 1990s methods of molecular genetics were used to determine the contributions of individual genes to different developmental processes, such as the segmentation of the Drosophila embryo [1]. However, during the past decade post-genome technologies have opened the door to identifying all of the genes engaged in such processes. In particular, collections of microarray data accumulated in public databases now cover a variety of different conditions and sometimes even the full life cycles for a range of evolutionarily distant species. These data provide new opportunities to identify complete ensembles of genes engaged in the specification of body plans and morphological diversification. Recently a number of such studies in different model systems have been conducted [2]–[9]. The initial reports as exemplified by [7], where the comparative analysis was extended across different phyla, provide evidence for the existence of deeply conserved co-regulated gene sets (kernels [10]) responsible for fundamental cellular functions. More recent studies focused on a set of fairly proximal yeast species have revealed that the regulation of even the most essential processes such as the cell cycle may not be conserved [2]–[4]. It was found instead that temporarily similar engagement of multi-component biological machines could be achieved by the species-specific regulation of different subunits within these complexes [11],[12]. Also, the regulation of genes, which are differentially regulated between species, was shown to be primarily driven by TATA-box containing promoters [5].

The aforementioned studies were done by “co-expression meta-analysis” extensively reviewed in [13] and in [14]. This method employs gene lists independently precompiled by condition-dependent clustering in individual species. The benefit of this approach is its ability to compare gene expression independently of species-specific experimental conditions. In contrast, “expression meta-analysis” [13] employs direct comparisons of the expression profiles of orthologue pairs. This approach can specifically pick up a condition or a time point when orthologous genes are differentially regulated between species. However, its use is restricted to a set of similar conditions, such as a time-series. This method requires data preprocessing, or in the case of temporal data, matching the corresponding time points, which due to differences in metabolism may not relate directly between the species. The computational frameworks as well as data sources for direct cross-species gene expression studies have only recently become available.

In a recent study we used datasets spanning several cell cycles of synchronized culture of fission and baker's yeast as a comparative training data source to develop a computational platform for expression meta-analysis [15]. Using so-called “time warping” [16]–[20] enhanced by noise suppression [15], we created alignment paths and successfully predicted the comparative duration of the cell cycle phases in baker's and fission yeast. In the present study we have employed the time warping method to compare mosquito and fruitfly embryos.

Thus far, the phylogenetic comparisons of gene expression have identified differential expression of orthologous genes implicated in similar processes. However, most such comparisons have been restricted to unicellular organisms [2]–[4] or to closely related multicellular species that manifest relatively few phenotypic differences. Moreover, such studies have examined excessively divergent species (yeast versus humans) [7], thereby complicating the association of discordant gene expression profiles with phenotypic variation.

We have selected divergent species that are nonetheless members of a common order of insects, the Diptera. We reasoned that mosquitoes and fruitflies possess a number of distinctive phenotypic traits, but are sufficiently similar to link such differences with orthologous clusters of discordantly expressed genes. These two flies belong to separate suborders (Nematocera and Brachycera) of Diptera and are thought to be separated by an evolutionary distance of ∼200 million years [21]. Mosquito and fruitfly larvae possess a number of striking differences, such as the representation of larval head, which is involuted in *Drosophila* but fully extended in mosquitoes. Another prominent feature, which was recently examined at the level of gene expression by our group [22],[23], is the presence in the mosquito embryo of a double-layered extraembryonic envelope (amnion and serosa), which is substituted by single amnioserosa in fruitflies (reviewed in [24]). We were interested in whether these and additional features could be detected by the comparative analysis of gene expression datasets.

The duration of embryogenesis in flies and mosquitoes differs significantly. At 25°C it takes ∼22 h for the fruit fly embryo and ∼50 h for the mosquito embryo to fully develop. For this reason, the temporal gene expression datasets for fly [25] and mosquito embryogenesis [22] were first aligned using time warping [15] and then analyzed for discordant gene expression. Since all the distinct developmental events taking place in the course of embryogenesis are expected to have specific timing and duration, the aim of the analysis was to cluster the orthologous gene pairs based on timing and duration of local discordances in their temporal expression profiles.

These studies reveal that a major gene cluster matches those known to be involved in the function of the mosquito serosa. This cluster is shown to be re-engaged later in development during cuticle synthesis. Significant discordances were also observed for a number of maternally expressed genes in flies and mosquitoes, consistent with the evolution of a sharp maternal-to-zygotic transition in gene expression higher Diptera. These studies provide a framework for the identification of the genetic circuits underlying embryonic diversity.

## Results

### Correspondence of Mosquito and Fruitfly Embryogenesis

Previous time-lapse microscopy [22] suggests that there is a simple linear correspondence in the embryonic development of fruitflies and mosquitoes, despite a ∼2-fold difference in duration (∼50 h at 25°C for *Anopheles* versus 24 h for the fruitfly). We used the time warping algorithm [15] to compare the Anopheles temporal microarray datasets and the available Drosophila dataset [25]. The term “time warping” is generally used to describe a set of computational procedures that allow matching the similar regions of numerical data, corresponding to the processes occurring at different time scales. *Anopheles* and *Drosophila* genomes are represented in genome databases by accordingly 12,604 and 13,781 annotated genes, most of which are represented on the corresponding microarray platforms (10,873 *Anopheles* genes and 13,056 *Drosophila* genes). Ortholog mapping (8,126 *Drosophila* genes matched to 8,047 *Anopheles* genes resulting in 10,708 orthologue pairs in ENSEMBL database) and ANOVA filtering (removing the genes showing no change in gene expression across the developmental time course) limited the total amount of data available for the time-warping and comparative gene expression analysis to 4,839 profile pairs, nearly 40% of all protein coding genes in the *Drosophila* genome (see Methods).

**Figure 1:**
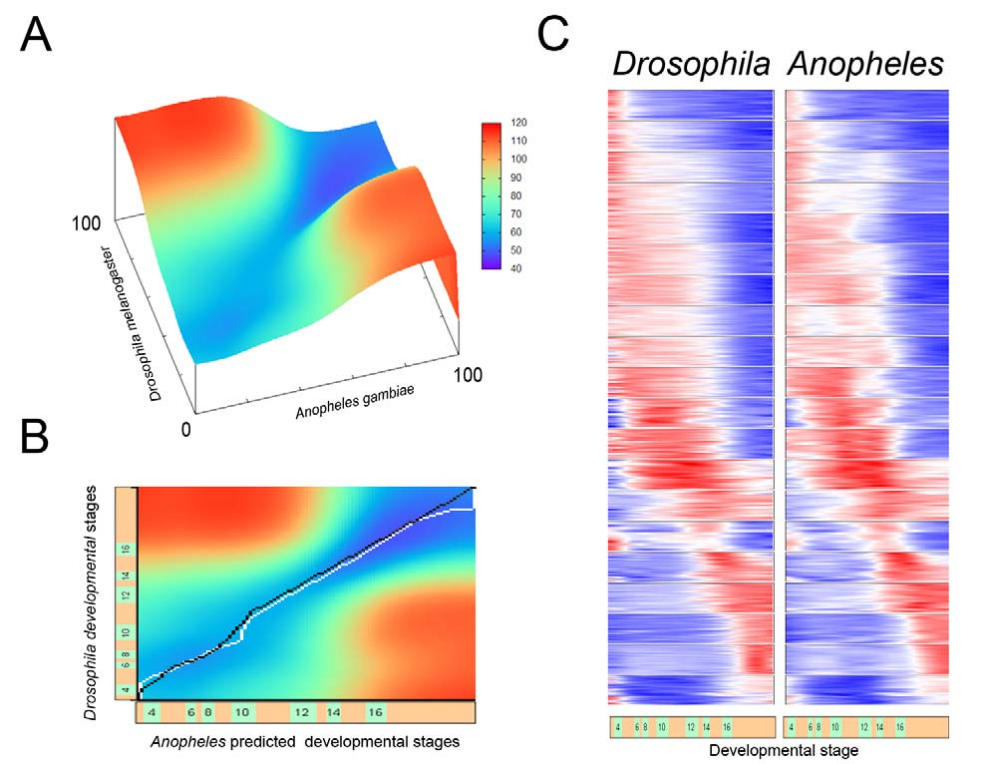
Linear correspondence between the fruitfly and the mosquito development. (A) Isometric view of the Pearson similarity matrix for normalized, log2- and z-transformed datasets, resampled to 100 points and Gaussian-smoothed in the window of 20 points (see Methods). *x*- and *y*-axis correspond to series of individual time points examined by gene expression screens in both species. Heatmap colors (blue, similar; red, dissimilar) correspond to numerical distance between the corresponding stretches of the datasets calculated by *Time-warp*. (B) 2D view of Pearson similarity matrix. White path indicates the automatic alignment (correspondence between the individual time-points in orthologous datasets) generated by time warping. Black path shows the correspondence between the sampling times in both organisms established on the assumption of linear correlation of developmental time. (C) Clusters of best correlating genes in both organisms after the transformation of datasets by global alignment. Under the cluster map is the diagram of the relative duration of *Drosophila* developmental stages [36].

Alignments of the Drosophila and Anopheles datasets suggest a near linear correspondence in embryonic development, despite the different rates of development (see Figure 1A,B and UCB web resource). There is a high level of concordance in the gene expression profiles: 1,172 of 4,839 profiles (24%) display a strong correlation (*r*>0.9), another 1,757 profiles (36%) exhibit a good correlation (0.9>*r*>0.6), and 744 pairs (15%) show a moderate correlation (0.6>*r*>0.3). Thus, ∼75% of the orthologous gene pairs exhibit very similar temporal profiles of expression in the divergent fly and mosquito embryos. Clustering the 1,538 most concordant expression profiles (based on *Drosophila melanogaster* expression data) reveal a striking correspondence during development (see Figure 1C and UC Berkeley web resource). The large, almost rectangular, regions of similarity in the beginning and in the end of the time courses but not in the middle (see the heatmap on Figure 1A,B) suggest that both organisms use related gene repertoires during late stages of embryogenesis that are considerably different from those used at earlier stages.

### Discovery of Discordant Gene Batteries

**Figure 2:**
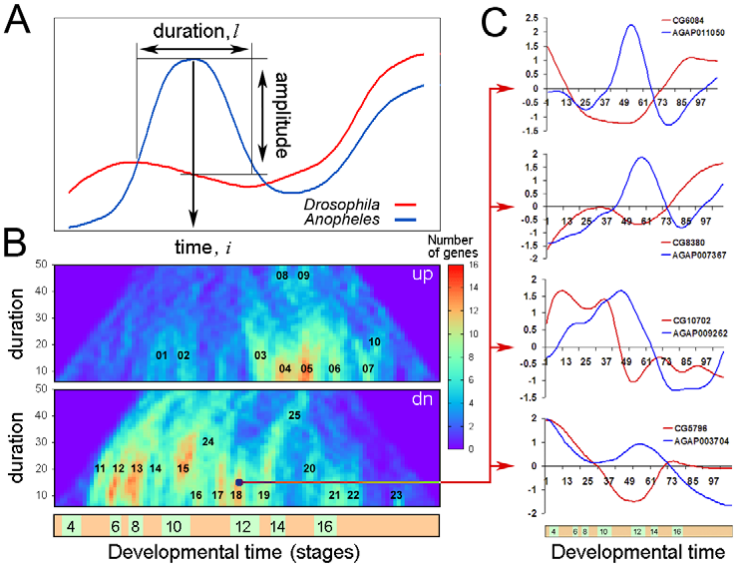
Representation of phylogenetic difference between the developmental gene expression datasets by two-dimensional heatmaps. (A) Schematic diagram of the strategy used for the evaluation of discordance in aligned temporal profiles of orthologous genes. The discordance was evaluated in a window of duration *l* at a time point *i*. The scoring function was designed to produce best scores when the amplitude of discordance was maximal inside and minimal outside the evaluated window. (B) Discordance heatmaps reflect the number of genes (shown by color intensity), which have a significant discordance (above cutoff) in expression profiles within a temporal window of a specific width (indicated by vertical axis) at specific relative developmental time point (shown by horizontal axis). The upper map contains the genes upregulated in the fruitfly embryo as compared to the mosquito. The lower map shows the genes upregulated in mosquito embryo compared to the fly. The heatmaps are set to account only for the discordances with the scores above the cutoff  = 1.7, which is the optimal cutoff for serosa detection. Distinct clusters of discordances with specific duration (window length) and the time of incidence are enumerated. The extracted gene lists are available for further analysis at UCB web resource. (C) Temporal expression profiles of orthologous gene pairs (blue, mosquito; red, fruitfly) with the highest discordance scores isolated from the cluster 18 on the heatmap in (B). Note the temporal co-incidence of the discordances on the expression profiles of these gene pairs.

We expected that the developmental events, which differ between the two species, would correlate with the discordant expression of numerous genes rather than just one or two. Moreover, we did not expect the individual members of such gene batteries to have similar expression profiles even within the same organism (Figure 2C), in contrast with previous models that assume similar expression of related genes [25]. To maximize the chance of identifying genes manifesting local as opposed to global differences in gene regulation, a scoring scheme was used that maximized discordance inside and the similarity outside the sliding window across the temporal axis of the comparative gene expression dataset (for details see Methods). The major parameters of this function are time (window position), duration of developmental event (window size), and the score (reflecting the amplitude of discordance). These parameters are sufficient to explicitly describe the pattern of discordance (Figure 2A).

For each relative time point and window length a list of genes was obtained with discordance scores above a chosen threshold. In this way, a comparison of the overall gene expression profiles between the two organisms could be represented as a two-dimensional heatmap, whereby the *x*-axis shows the relative developmental time and the *y*-axis indicates the window length and the color corresponds to the number of the genes with a similar discordance pattern (Figure 2B). The map patterns reveal territories (gene clusters) corresponding to groups of discordant genes, which exhibit variability at a specific time-point in development. This map could be constructed for any arbitrarily chosen discordance cutoff. We therefore went further to check whether these clusters might represent evolutionary innovations (e.g., distinct morphological structures) involving the coincident deployment of a large set of genes in one of the organisms.

### Detection of Serosa Gene Batteries

**Figure 3:**
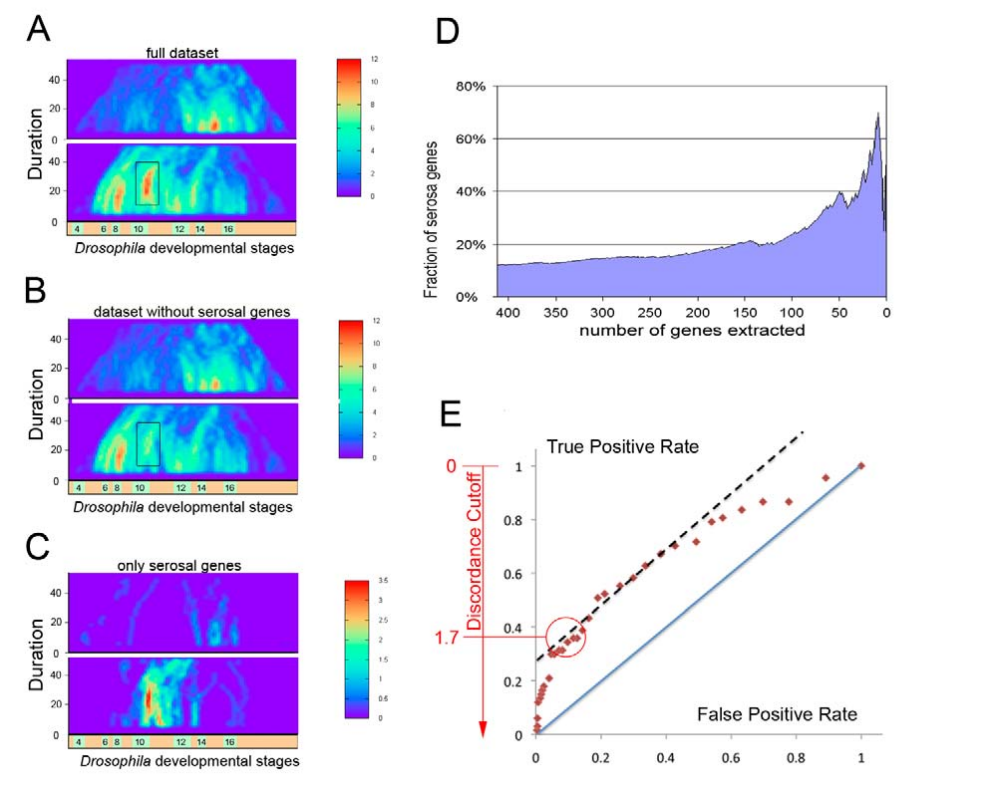
Clusters on the discordance map reflect the presence of new cell types in one of the organisms. (A) Discordance heatmaps for mosquito versus fruitfly development (discordance cutoff  = 1.7). Horizontal and vertical axis correspond to relative temporal position and length of the window used for discordance analysis. The color corresponds to the number of orthologous gene pairs within this window with discordance values above the cutoff. (B) Discordance heatmaps for the datasets without the mosquito serosal genes (as defined in [22]) and their fruitfly orthologues. Note the disappearance of the cluster 15 (marked by transparent black rectangle around the cluster) on the map of the genes upregulated in mosquito. (C) Discordance heatmaps constructed for the datasets made exclusively from the mosquito serosal genes (taken from [22]) and their fruitfly orthologues. (D) Enrichment of the serosal genes in the ranked gene list extracted from cluster 15 at different discordance cutoffs (from 0 to 3). Small cutoffs (<1) produced large gene lists (>100 genes) with small enrichments (<20%), and large cutoffs (>1.7) produced small gene clusters (<25 genes) with good enrichment (>30%). (E) Receiver Operating Characteristic (ROC) curve for ranked gene lists extracted from cluster 15 at a range of discordance cutoffs (from 0 to 3).

We used the dissimilarity in the structure of extraembryonic membranes of *Anopheles* and *Drosophila*, investigated in our prior study [22], namely the absence of the serosa in the fruitfly embryo, as a model of differentially represented trait/organ/tissue type. To determine whether any of the major hot spots (gene clusters) on the discordance heatmap corresponds to serosa, we built the maps selectively for the serosal genes (249 genes expressed higher in serosa than in the *embryo proper* with Log2Fold >0.7; see [22]) (Figure 3C) as well as for the whole dataset with (Figure 3A) or without the serosal genes (Figure 3B). Taken separately, the serosal genes manifest a prominent cluster at the position corresponding approximately to 12 h of mosquito development and 6 h of fruitfly development. While the general discordance map pattern remained the same, the depletion of the serosal genes from the dataset resulted in the disappearance of this cluster (cluster 15, see the UCB web resource) from the map (Figure 3B). On the example of the serosal cluster, we therefore conclude that the hotspots on the discordance heatmap may correspond to conspicuous or cryptic differences in anatomy or regulatory programs employed during development.

We further used the serosal cluster to define the best discordance cutoff that would be applicable for a wide range of morphological differences. Specifically, we composed a training set of positives (serosal genes) and negatives (non-serosal genes) from the discordant genes that could be extracted from the cluster 15 region at discordance cutoff  = 0 and built the Receiver Operator Characteristic (ROC; see Figure 3E), which describes the True Positive Rate (TPR) and False Positive Rate (FPR) of this training set within the gene sets extracted from the cluster 15 at different discordance cutoffs (0 to 4). Using the classic analysis of the ROC curve we identified the best discordance cutoff value, as the one, which corresponds to the point when the number of true positives among the genes that would augment the list, if the cutoff was further increased, is higher than the number of false negatives. Practically it is the point on the ROC curve when the first derivative of True Positive Rate as a function of False Positive Rate rises above 1 (TPR∼0.4, FPR∼0.2 matching the discordance cutoff 1.7; Figure 3E). The 1.7 cutoff represents a compromise between the sensitivity and the specificity of detection, resulting in 30% of serosal within the 102 genes of the cluster. Yet at higher cutoffs (2.9) further increase in specificity (8 serosal out of 12 total genes) despite a significant loss in sensitivity could be achieved (Figure 3D). The clusters extracted from the discordance heatmaps can be further processed by standard genomics tools, such as GO enrichment analysis (see below).

### Maternal Transcripts

The successful identification of the serosal genes led us to analyze additional discordant gene clusters. There are about 23 discordant hotspots (Figure 2B). Functional assignment of the newly identified clusters was investigated using FlyBase GO terms and controlled vocabulary annotations from the BDGP in situ database (see UCB web resource for annotations).

**Figure 4:**
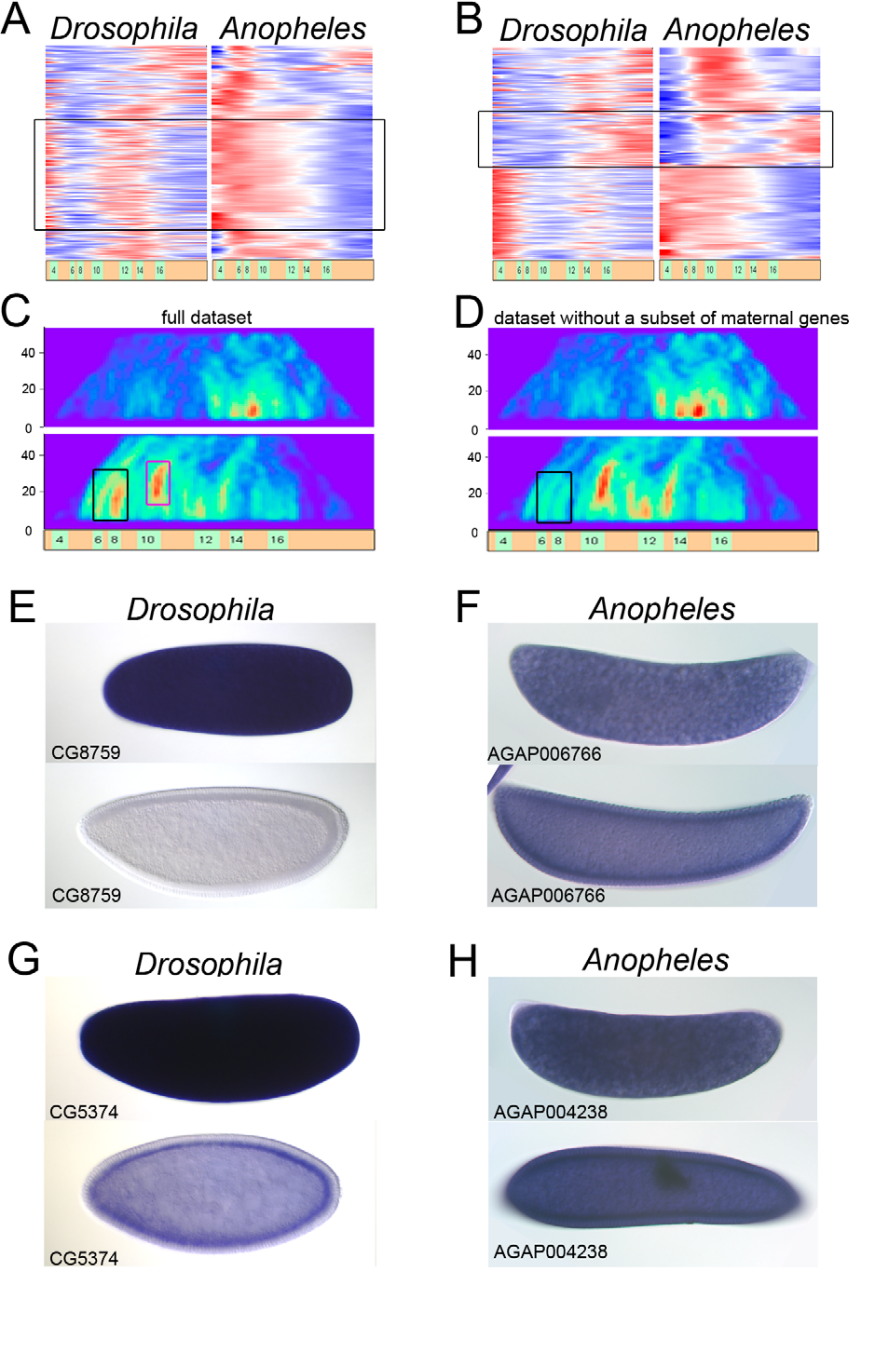
Functionally related and co-regulated genes are revealed by the temporal re-engagement pattern. (A) Clustered expression profiles of genes extracted from clusters 12 and 13 at low discordance cutoff  = 1 with Glob-mapper program. A battery of maternal genes (marked with black rectangle), which is expressed persistently in mosquito, is inhibited at gastrulation in *Drosophila* and re-expressed again later. The area of the heatmap (duration  =  [5–28], position  = [15–25] ) used for extraction is also marked with black rectangle on (C). (B) Clustered expression profiles of genes extracted at discordance cutoff  = 1 from area covering cluster 15. The area (duration  =  [2–28], position  =  [34–39]) used for extraction is marked with magenta rectangle on (C). The double-engaged gene set marked by black contains significant number of serosal genes. (C) Discordance heatmaps for the full dataset representing the mosquito versus fruitfly development (discordance cutoff  = 1.9) Horizontal and vertical axis correspond to relative temporal position and length of the window used for discordance analysis. The color corresponds to the number of orthologous gene pairs within this window with discordance values above the cutoff. (D) Discordance heatmaps for the datasets without the battery genes selected with black rectangle in (A). Note the disappearance of clusters 12 and 13 (marked by black rectangular contour around the territory meant to be occupied by the clusters). (E,F and G,H) The mosquito and corresponding fruitfly examples of the ortholog gene pairs (AGAP006766-CG8759 and AGAP004238-CG5374) from the battery of double engaged maternal genes marked with green rectangle in (A). Upper panels correspond to early nuclear divisions reflecting maternal distribution of the transcript; lower panels show the distribution of the transcript at developmental stage close to gastrulation in both organisms.

One of the discordant clusters (clusters 12–13, see Figure 2B and UCB web resource) corresponds to a set of maternal genes in both flies and mosquitoes. In flies, these genes exhibit a sharp reduction in expression during the maternal to zygotic transition but display continuous expression in mosquito embryos (Figure 4A). RNA in situ hybridization assays (Figure 4E,F, and G,H) show that these genes are ubiquitously expressed. In *Drosophila* these transcripts are rapidly lost at the onset of gastrulation, while in *Anopheles* they persist throughout the periods of gastrulation and germband elongation without significant changes in levels (Figure 4E,F, and G,H top versus bottom panels). Removing the maternal gene battery from the comparative datasets revealed that it indeed corresponds to clusters 12 and 13 on the discordance heatmap (Figure 4C,D).

**Figure 5:**
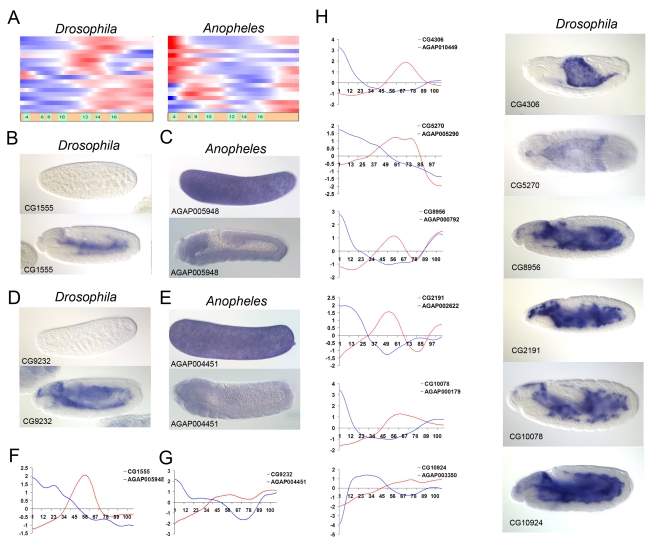
A set of Drosophila genes expressed in the yolk is maternal in mosquito. (A) Temporal expression patterns of a set of genes, which are co-expressed at mid-embryogenesis in Drosophila and are coincidently maternal in mosquito. (B,C) Example of an ortholog pair (AGAP005948-CG1555) from (A) analyzed by RNA situ hybridization. Top panels correspond to freshly laid eggs; bottom panel corresponds to mid-embryogenesis. (D,E) Another example of an ortholog pair from (A) (AGAP004451-CG9232). Top panels correspond to freshly laid eggs; bottom panel corresponds to mid-embryogenesis. (F and G) Line graph representation of genes analyzed in (B,C) and (D,E) (expression profiles of mosquito genes are in blue; the fruitfly genes are in red). (H) Other examples of genes from (A), only the fruit fly RNA in situ hybridizations are shown.

Another unique cluster (cluster 8, see Figure 2B) was enriched for genes expressed in yolk in *Drosophila*. Among the genes expressed in the yolk we noticed another example of coordinate difference in gene expression. A number of metabolic genes such as CG9232 (galactose metabolic process) and others expressed in *Drosophila* yolk at mid-embryogenesis show maternal expression in mosquito, suggesting that the dynamics of yolk metabolism differs dramatically between the two branches of Diptera represented by these insects (Figure 5A–H).

## Discussion

This study provides a conceptual and computational framework for cross-organismal temporal alignments and comparisons of species-specific transcriptional datasets. The resulting heatmaps of discordant gene clusters identified distinctive patterning properties in Dipteran embryos, including the organization of extra-embryonic tissues and the maternal-zygotic transition (see Figure 2B and UCB web resource). There is surprising concordance in the development of Drosophila and Anopheles embryos, despite different rates of growth and distinctive patterning features. The heatmaps and alignments of gene expression datasets (representing more than 4,000 orthologous gene pairs) obtained with the time warping algorithm suggest a near linear relationship in their embryonic development. Interestingly the alignment path manifested a subtle deviation from linearity at stage 9, presumably shortly after serosa completion in Anopheles (Figure 1B). The removal of serosal genes from the datasets did not result in change in the automatic alignment path, suggesting that this time point may indeed represent temporarily local slow down of gene expression programs in *Anopheles embryo proper* compared to *Drosophila*.

The heatmaps obtained with the discordance mining algorithms implemented in *Peak-mapper* and *Glob-mapper* identify clusters of discordant genes that represent either unique tissue types (such as serosa) or changes in regulatory pathways such as that governing the turnover of maternal transcripts. The percentage of false positive genes (as judged by the analysis of the serosa gene cluster) was expected to be relatively high. One approach to this problem is the application of annotation methods such as GO-term enrichment or controlled vocabulary annotations (see UCB web resource).

“Gene sharing” or “co-option” manifested in redeployment of gene batteries in a diverse set of tissues is a recurrent theme in the evolution of animal embryos [26]. One of the mechanisms for gene co-option, specifically when it happens in the absence of gene duplication, is the diversification of regulatory regions resulting in acquisition of new territories of expression. For example, the skeletogenic mesoderm of the sea urchin embryo employs a conserved gene battery that is used for the secretion of the adult exoskeleton [27],[28]. Previous studies showed that the serosa gene battery is reengaged later in development, during the time of embryonic cuticle production [22]. The fact that an identical gene set is used twice during development at different time points or in different tissues suggests that the genes don't fall within this set due to random co-occurrence. In fact this reengagement is observed due to similarities of the cuticle secreted by the serosa at early stages [29]–[32] and the embryonic cuticle produced at the end of embryogenesis. We propose that the incidence of gene battery reengagement might be used as a further indicator of functional relevance of the gene within the battery or otherwise a filter that could remove the false positives from the genes extracted from the discordance cluster. Indeed, serosa genes can be identified by their biphasic expression in the mosquito as compared with the single late peak of expression in the fruitfly embryo (Figure 4B). Perhaps the low threshold discordance clustering in combination with verification of the reengagement patterns can be used as an alternative strategy for the extraction of functional gene sets from comparative datasets.

Previous whole-genome comparisons focused on ancestral gene networks based on phylogenetic conservation of gene expression patterns. Here, evidence was presented that discordant clusters provide a means for identifying gene batteries involved in evolutionary diversification and novelty. This approach was used to identify *de novo* the changes in the turnover of maternal transcripts in the *Drosophila* and *Anopheles* embryos and in temporal expression of a subset of yolk genes. The rapid turnover of maternal genes correlates with increased rates of embryogenesis in higher Dipterans.

## Materials and Methods

### Mosquito Stocks and Egg Collection

*Anopheles gambiae* population was reared at 27°C, 75% humidity, with a 12-h light/dark cycle. Adults were maintained on a 10% sucrose solution and females were blood-fed on anesthetized hamsters. For synchronized embryo collection the females were placed in the dark at 27°C for 1 h inside a 15 cm petri dish lined up with circles of wet Whatman paper. The developmental time was counted starting from the moment the Whatman paper was moisturized with water. Mosquito embryo fertilization happens at the moment of egg laying. Due to constraints in the experimental setup, after 2 h following the start of collection, the eggs were shifted from 27°C and constantly kept at 25°C.

### Cross-Species Microarray Data Analysis

Drosophila-Anopheles ortholog pairs were downloaded using BIOMART interface of ENSEMBL website. Drosophila developmental microarray data-course was obtained from [25]. Mosquito developmental data-course (http://www.ncbi.nlm.nih.gov/geo/, accession number GSE15001) was taken from our prior studies [22]. Low level microarray data treatment involved standard quantile normalization of microarray data in log2 space [33] and Z-score normalization of time points (Gene_expr_value-mean(across development))/stdev(across development)) [33]. Both *Drosophila* and *Anopheles* datasets were filtered based on variation between biological triplicate's points using standard ANOVA analysis with very mild thresholds (*p*<0.05). In cases when several probesets were corresponding to a single gene, a probeset with a better (smaller) ANOVA *p* value (i.e., more consistent change within replicates and higher change across the time points) was chosen. In addition, the *Drosophila* expression array data [25] was filtered based on correlation with the *Drosophila* tiling array data [34] with mild thresholds as well (*r*>0.25). Finally, the expression profiles were superimposed based on the table of orthologs taken from ENSEMBL. Profiles corresponding to genes with multiple orthologs (paralogues) were multiplied, where necessary. These procedures produced 4,839 profile pairs, corresponding to 4,072 unique *Drosophila* genes.

### Construction of Global Alignment

Global alignment was constructed based on Kruskal-Liberman time warping algorithm [16],[35] as described in our previous study [15]. In brief, using the *RZ-smooth* filter from our software package (see web resource), the microarray data were resampled to 100 points in each dataset and smoothened using Gaussian function with a standard deviation corresponding to ∼2 original time points (15 relative points in resampled datasets). To compensate for the impact of terminal regions, after the resampling both datasets were truncated for less than 1 original time point at the beginning and the end. Similarity matrices and the corresponding global alignment path based on resampled and truncated datasets were constructed using time-warping algorithm. Specifically, local uncentered Pearson correlation between two sliding windows of *l* (*l* can be any natural number, we used *l*  = 20) points each was calculated. This method, which was introduced in [15], is less sensitive to the interspecific noise in the data and better captures the subtle similarities between the datasets. Following the alignment, datasets were smoothened once more (using half of the original smoothing window) to compensate for step-like patterns in alignment curve.

### Identification of Concordant and Discordant Gene Pairs

Concordant genes were identified in the aligned datasets based on the global uncentered Pearson correlation. For the identification of the discordant genes, for each orthologous pair of resampled and aligned expression profiles the sums of square differences *F* (between the matching points on profiles) were calculated for the time point *i* and window length *l*—inside (*F^win^*) and outside of the window (*F^ext^*). Conditional probabilities *p* were computed based on distribution of the *F* values in the datasets A and B (for all genes). *α* is a pseudocount, limiting the probability values (α = 0.01 was used in this study).
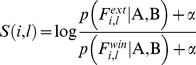
(1)


For every orthologous gene pair the discordance score *S*(*i*, *l*) was calculated as a function of *i* (window position) and window length (duration) *l*.

For any single comparison two heatmaps representing upregulated and downregulated genes (with reference to expression in *Drosophila*) were built. Batteries of discordant genes were identified as “hot spots” in the (*i, l*) parameter space, showing high numbers of genes with scores exceeding a threshold (*S*>1.5) for a given set of parameters *i*, *l*. For the further analysis, such as evaluation of annotation enrichment in the local discordance clusters (hotspots), we extracted the genes from an arbitrarily defined region (*i*1–*i*2, *l*1–*l*2) on the map, corresponding to the hotspot.

### Software

Different stages of dataset processing as well as the detailed help for the programs can be downloaded from http://flydev.berkeley.edu/cgi-bin/GTEM/dmap_dm-ag/index_dmap.htm. In short, initial dataset parsing, specifically the resampling and smoothing, were accomplished by *RZ-smooth*. The similarity matrices and alignment paths were built by *Time-warp*. At the next step the datasets were matched by *M-align* and a database of discordances was built by *Peak-mapper*. Finally the threshold-dependent heatmaps were constructed by *Glob-mapper*, which as part of its interface also allowed extracting the content of the discordance clusters (hotspots).

### Whole-Mount In Situ Hybridization

The images of whole-mount in situ hybridizations of *Drosophila* embryos were taken from BDGP in situ database (http://www.fruitfly.org/cgi-bin/ex/insitu.pl). Mosquito embryos were collected and fixed as described previously [23]. The hybridization dig-labeled anti-sense RNA probes against specific *A. gambiae* genes were generated by RT-PCR amplification from embryonic RNA and reverse transcription. A 26 bp tail encoding the T7 RNA polymerase promoter (TAATACGACTCACTATAGGGAGA) was included on the 5′ side of the reverse primer.
